# Validation of two techniques for intraoperative hyperspectral human tissue determination

**DOI:** 10.1117/1.JMI.7.6.065001

**Published:** 2020-11-19

**Authors:** Eric L. Wisotzky, Benjamin Kossack, Florian C. Uecker, Philipp Arens, Anna Hilsmann, Peter Eisert

**Affiliations:** a Fraunhofer Heinrich Hertz Institute, Computer Vision and Graphics Group, Berlin, Germany; b Humboldt-Universität zu Berlin, Visual Computing Group, Berlin, Germany; c Charité—Universitätsmedizin Berlin, Department of Otorhinolaryngology, Berlin, Germany

**Keywords:** hyperspectral imaging, multispectral imaging, snapshot camera, filter-wheel setup, calibration, human soft tissue, tissue analysis

## Abstract

**Purpose:** Hyperspectral imaging (HSI) is a non-contact optical imaging technique with the potential to serve as an intraoperative computer-aided diagnostic tool. Our work analyzes the optical properties of visible structures in the surgical field for automatic tissue categorization.

**Approach:** Building an HSI-based computer-aided tissue analysis system requires accurate ground truth and validation of optical soft tissue properties as these show large variability. We introduce and validate two different hyperspectral intraoperative imaging setups and their use for the analysis of optical tissue properties. First, we present an improved multispectral filter-wheel setup integrated into a fully digital microscope. Second, we present a novel setup of two hyperspectral snapshot cameras for intraoperative usage. Both setups are operating in the spectral range of 400 up to 975 nm. They are calibrated and validated using the same database and calibration set.

**Results:** For validation, a color chart with 18 well-defined color spectra in the visual range is analyzed. Thus the results acquired with both settings become transferable and comparable to each other as well as between different interventions. On patient data of two different otorhinolaryngology procedures, we analyze the optical behaviors of different soft tissues and show a visualization of such different spectral information.

**Conclusion:** The introduced calibration pipeline for different HSI setups allows comparison between all acquired spectral information. Clinical *in vivo* data underline the potential of HSI as an intraoperative diagnostic tool and the clinical usability of both introduced setups. Thereby, we demonstrate their feasibility for the *in vivo* analysis and categorization of different human soft tissues.

## 1 Introduction

Pathological structures, e.g., tumors, can develop between or in direct proximity to important structures, e.g., nerves. To reach the operating area, the surgeon has to expose the nerves without causing substantial damage.^1^ This structure exposition process is of high risk and difficult, because damage to healthy structures, like nerves, can cause a temporary or permanent paralysis of the affected region. Furthermore, in normal light conditions, the different pathological tissue types are nearly undifferentiable from healthy neighboring tissue and the detailed anatomy differs widely between each patient. Therefore, it is very time-consuming to prepare important tissue without damaging the healthy anatomical structures,^2^ but this slow and careful process is an important part of the operation. An automatic analysis, differentiation, and visualization of important tissue structures would allow the surgeon to perform operations faster and with lower risk.

Hyperspectral imaging (HSI) is established in biomedicine for cell segmentation and skin analysis as it shows great potential to derive information about the biophysical and biochemical parameters.^3^^,^^4^ It has been shown that different tissue types show different optical characteristics in terms of reflection, transmission, and absorption for different wavelengths.^5^ Different modalities exist to acquire a hyperspectral data cube. One possibility is to use different filters in front of the sensor or behind the light source, which sequentially step through the hyperspectral space (filter-wheel setup).^6^^–^^8^ Such a filter-wheel setup requires time to capture all filter responses and thereby the spectral space. In contrast to the filter-wheel setup, snapshot hyperspectral cameras can be used to acquire the complete data cube at once, though with lower resolution.^9^ Thus there are two ways to achieve a spectral partition: wavelength selection on the illumination side and wavelength selection on the detector side. Both possibilities hold different physical and technical properties and without a careful and accurate calibration process, the different properties of both approaches result in incomparable data.

Several HSI systems exist in biomedicine, e.g., hyperspectral snapshot cameras, used in retinal imaging^10^^,^^11^ and human cortex imaging,^12^ as well as filter-wheel setups, used in colorectal tumor imaging.^13^ HSI for image-guided tissue differentiation is still part of extensive research activities.^3^^,^^14^ In order to exploit HSI for image-guided tissue differentiation, extensive knowledge about the technical system and the investigated tissue types is important. On the one hand, the optical properties of human soft tissue show large variability. On the other hand, the measured raw hyperspectral data cube contains pixel values within each spectral band, which do not necessarily represent the real reflectance of the scanned area. Artifacts of the optics and electronic components influence the measured signals and have to be corrected.^15^^,^^16^ Further, as described by Aasen and Bolten,^17^ differences in the illumination settings have an influence on the calibration process and the data processing. Finally, the measured signal has to be transformed into a physical system-independent value, e.g., reflectance, to make the data comparable among different settings and useful for signal processing.^18^ These aspects make a robust calibration process necessary for potential clinical practice.^19^^,^^20^

This work presents a calibration pipeline for different possible hyperspectral setup configurations addressing the above-mentioned challenges. We describe two approaches for intraoperative image-guided soft tissue analysis, the first selects the wavelengths on the illumination side and the second on the sensor side and introduce our robust calibration pipeline, which makes both systems comparable to each other.^21^ Both approaches are validated using a color chart with 24 different well-defined and calibrated spectral tiles^22^ as reference spectra. In addition, the resulting data become comparable to data acquired with other spectroscopic analysis methods, e.g., spectrograph. The calibration information provided by the filter and camera manufacturers is validated by analyzing objects with a well-known spectrum. Identified variances between measured and reference data are used to adapt the calibration chain for clinical *in vivo* analysis. First, clinical results are presented to demonstrate the proposed different optical tissue behaviors and to show the feasibility of intraoperative optical tissue differentiation. This is opening the possibility to present additional intraoperative tissue information to the surgeon.

## 2 Methods

**Fig. 1:**
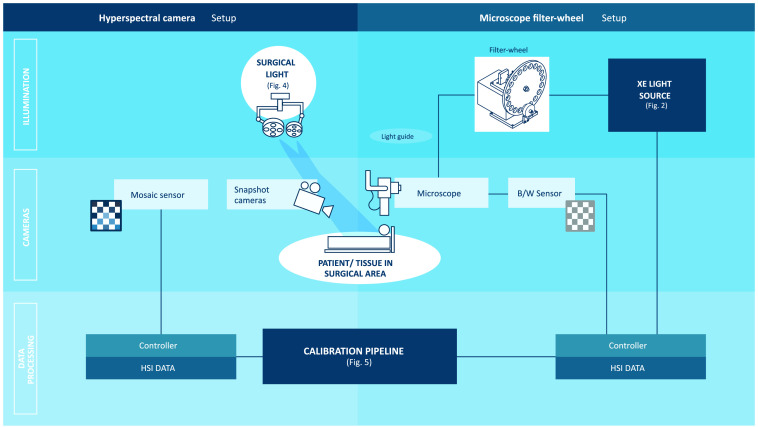
Both setups, hyperspectral camera setup (left part) and microscope filter-wheel setup (right part), are sketched. Each setup can be divided into three parts: illumination unit (top row), camera and sensors (middle row), and data processing unit (bottom row).

In the following, the two setups used in this study are presented. As overview, a diagram describing both systems can be found in Fig. 1. Both systems can be split into three parts: illumination unit, camera and sensor, and data processing unit.

### 2.1 Microscope Filter-Wheel Setup

**Fig. 2:**
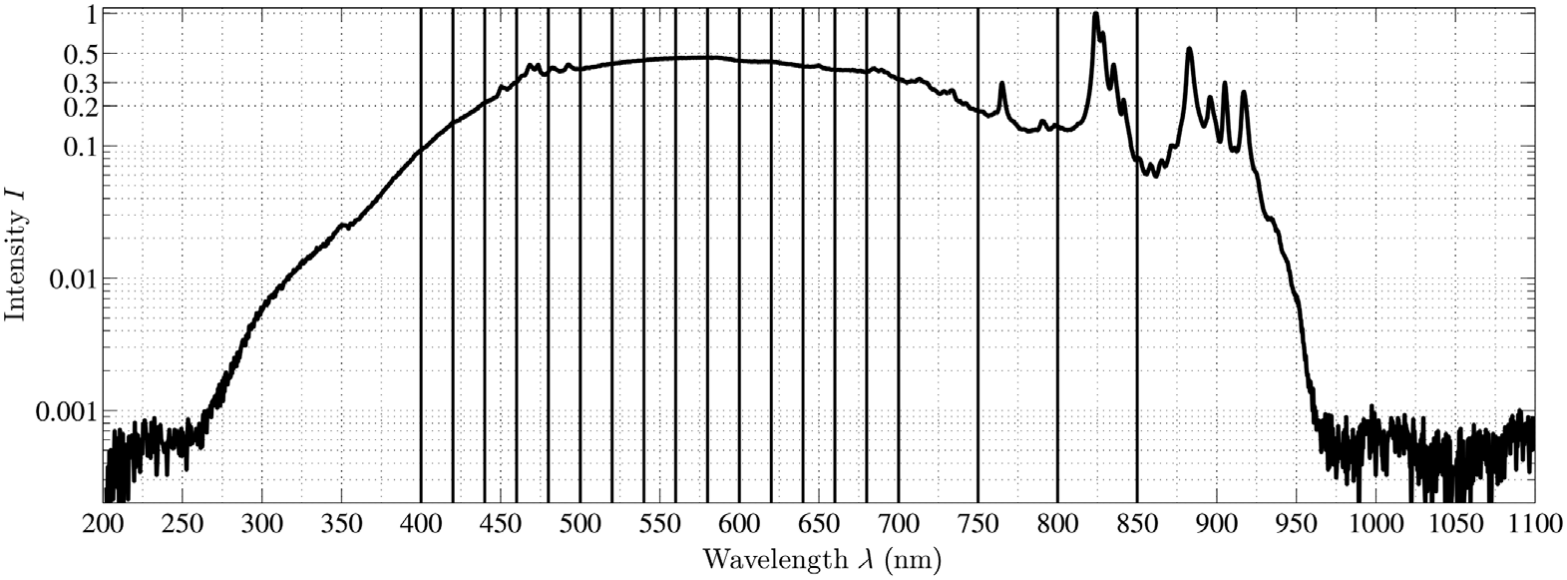
The spectrum of the 175-W xenon light source used in our setup. The relevant interval for illumination is 400 to 850 nm. In this interval, specific wavelengths are selected (solid vertical lines for the microscope filter-wheel setup) for illumination such that the illumination intensity stays almost constant and intensity peaks (e.g., between 810 and 845 nm) are skipped.

We build upon the digital microscope filter-wheel setup presented by Wisotzky et al.^23^^,^^24^ and change the illumination unit from an LED source with its challenging inhomogeneous spectrum to a 175-W xenon (Xe) light source of LEJ GmbH, Germany. Compared to the previously used LED source, the Xe light exhibits a wider and brighter spectrum in the interval of interest from 400 to 850 nm, plotted in Fig. 2. Additionally, the internal DC supply of the Xe source reduces time-based fluctuations of the luminous flux, which allows quantitative long-term microscopic measurements. The luminous flux typically lies at ∼500 lumen and the radiometric power of ∼1  mW/nm is nearly homogeneous in the spectral range of interest. As this is a low level of intensity, during the data acquisition all other light sources, e.g., ceiling and operation light, in the room must be turned off to avoid scattered and indirect light from these sources.

Further, we expand the multispectral range from 16 to 19 channels. To the 16 channels in the visual spectrum [400 to 700 nm, with a step size of 20 nm and full-width at half-maximum (FWHM) of 10 nm], we add three channels in the near-infrared (NIR) spectrum (750, 800, and 850 nm). These channels have been chosen because of the wider homogenous spectrum and to avoid the high peak between 800 and 850 nm, see Fig. 2. Each of these three filters has FWHM of 25 nm, see Table 1.

**Table 1:** The spectral peak and FWHM of each band for both setups and all used cameras and filters. The bands are sorted, starting with lowest λ, and the numeration does not correspond to the index positions on the sensor.

	Band #	0	1	2	3	4	5	6	7	8	9	10	11	12
Filter-wheel	λ (nm)	400	420	440	460	480	500	520	540	560	580	600	620	640
	FWHM	10	10	10	10	10	10	10	10	10	10	10	10	10
4×4 camera	λ (nm)	463	471	478	489	490	504	518	531	543	567	580	592	603
	FWHM	10.8	15.5	20.2	10.8	12.1	10.8	9.4	7.4	7.4	7.4	7.4	7.4	10.8
5×5 camera	λ (nm)	693	707	732	746	758	772	784	798	809	821	839	851	861
	FWHM	4.0	4.4	5.1	4.7	7.1	6.8	6.8	8.5	6.8	7.1	10.1	8.1	8.5
	Band #	13	14	15	16	17	18	19	20	21	22	23	24	
Filter-wheel	λ [nm]	660	680	700	750	800	850							
	FWHM	10	10	10	25	25	25							
4×4 camera	λ (nm)	616	626	638										
	FWHM	13.4	15.5	15.5										
5×5 camera	λ (nm)	872	881	891	900	910	924	932	940	948	955	961	966	
	FWHM	8.8	10.9	12.2	15.0	12.9	15.3	14.2	18.7	19.4	25.0	17.7	15.3	

The sensor and optics specifications remain the same as presented by Wisotzky et al.^23^ The sensor output resolution is 1920×1080  pixels and the used focal length at maximum zoom is 65 mm. The working distance (WD) is about 210 mm.

### 2.2 Hyperspectral Camera Setup

**Fig. 3:**
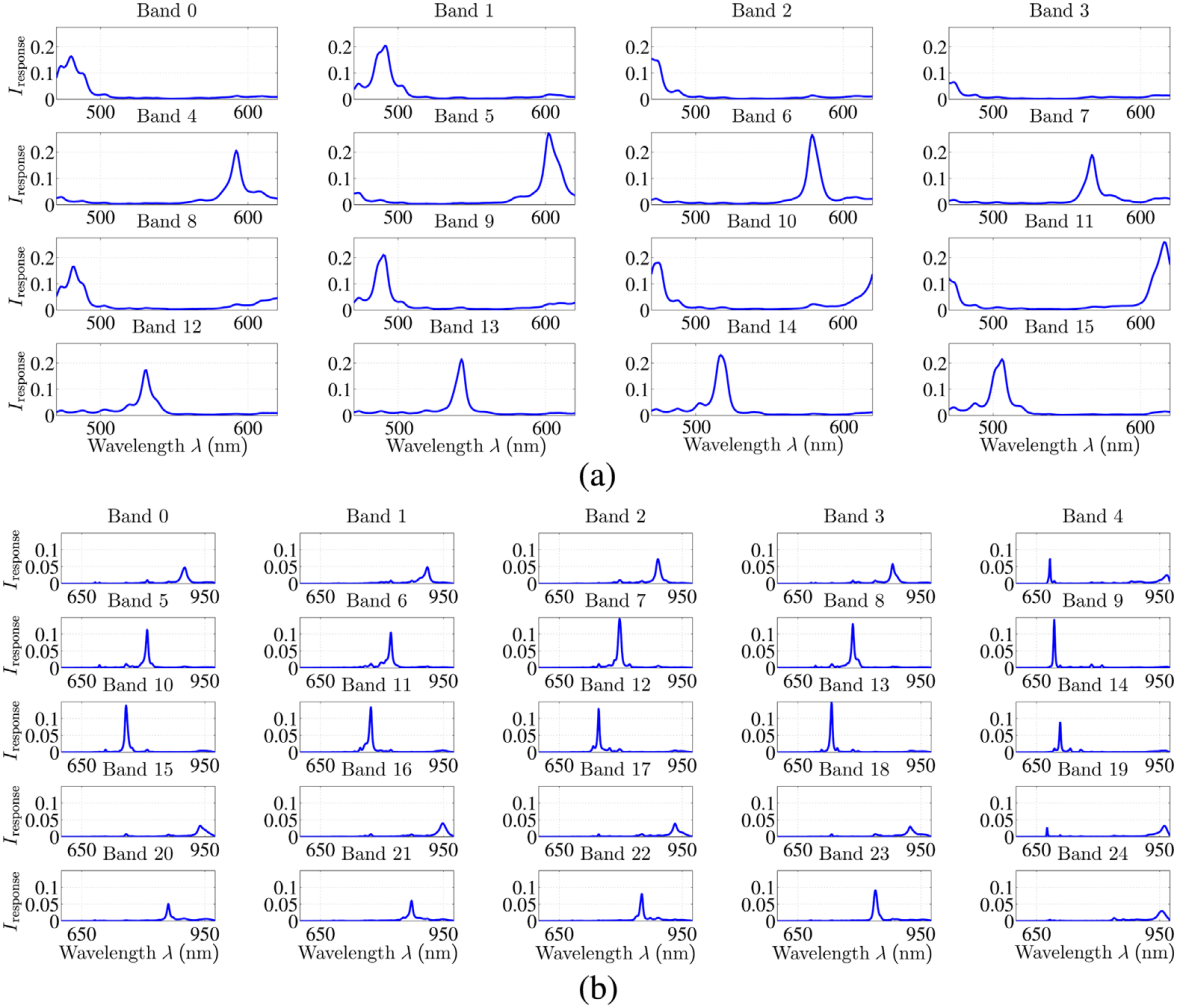
The specific sensor responses for (a) all 16 bands of the 4×4 spectral pattern of the used visual hyperspectral snapshot camera and (b) all 25 bands of the 5×5 spectral pattern of the used NIR hyperspectral snapshot camera presented by the manufacturer XIMEA, Germany.

The alternative HSI setup consists of up to two hyperspectral snapshot cameras of XIMEA, Germany. A snapshot camera has the advantage of acquiring the complete hyperspectral dataset in a single shot, whereas on the other hand, the spatial resolution is reduced due to the larger filter array. The first camera holds a 4×4-filter array, resulting in 16 HSI-bands between 460 to 630 nm with different FWHMs, from FWHM=4.69  nm at λ=460  nm in band 7 up to FWHM=20.18  nm at λ=478  nm in band 0, cf., Table 1. The second camera holds a 5×5-filter array, sensitive from 600 to 975 nm. Since the filters of the camera show two main peak responses in some pixels of the 5×5-filter array, a 675-nm long-pass filter is placed in front of the camera, resulting in 25 HSI-bands between 675 to 975 nm with an FWHM range from FWHM=3.99  nm at λ=693  nm in band 9 up to FWHM=25.00  nm at λ=955  nm in band 25, cf., Table 1. Each band has a specific response pattern containing primary and eventually secondary peaks in the sensitive interval of the sensor, see Fig. 3. Therefore, the band response is not a correct spectral signature as the signal is a combination of the sensor level response and the system level components. To measure a correct spectral signature, it is required to calibrate the signal through spectral correction.^25^ Further, the exposure times for both hyperspectral cameras will differ due to different sensor response characteristics. For the 4×4 HSI camera, the peak response intensity for each band is about 20%, except band 3 with ∼10%, see Fig. 3(a). For the 5×5 HSI camera, the peak response intensity for each band is lower with about 10%, see Fig. 3(b). Hence, the exposure time has to be longer for the 5×5 HSI camera to achieve a similar sensor response. These response gaps of the sensors, i.e., response intensity difference between band 3 and all others of the 4×4 sensor as well as bands 0 to 4 and bands 15 to 24 on one side and bands 5 to 14 on the other side of the 5×5 sensor will be addressed in the calibration process.

**Fig. 4:**
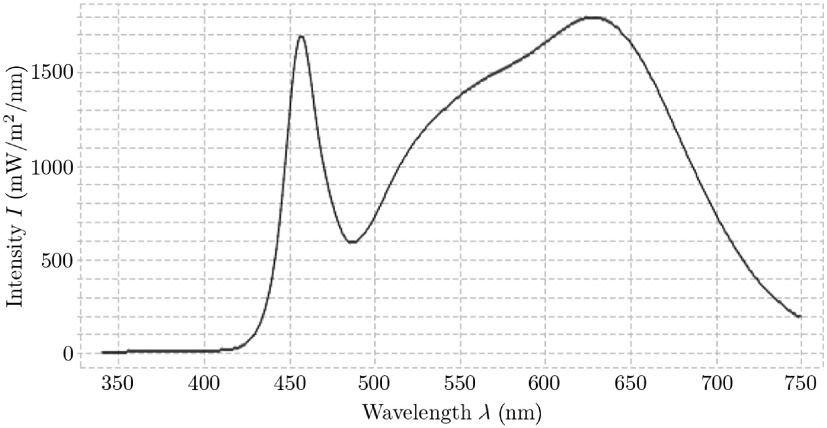
The spectrum of the surgical LED light source used in our setup. Compared to the used Xe source, cf., Fig. 2, this LED spectrum is very inhomogeneous, especially the intensity is decreasing rapidly starting at 680 nm, which results in low illumination intensity for the NIR spectrum.

The sensors of both cameras have a resolution of 2048×1088  pixels, in which the filter area is 2048×1024  pixels for the 4×4 HSI camera and 2045×1080  pixels for the 5×5 HSI camera. Both cameras hold a 75-mm f/2.8 lens and are aligned on a rack, focusing on the same point in a distance of ∼250  mm, which is the smallest possible WD for the setup and thus, the WD of the hyperspectral camera setup. We use two cameras to increase the number of spectral channels over the complete visual and NIR range (465 to 975 nm) while keeping a reasonable resolution. We tested two configurations for scene illumination, the Xe source of the microscope filter-wheel setup (in a distance of ∼210  mm) and the surgical LED light, see Fig. 4, in a distance of ∼300  mm. The large difference of the luminous flux of the LED light (which is much higher) and the Xe source make a robust calibration of the complete system indispensable. Additionally, the intensity spectrum of the LED source is inhomogeneous with a high peak at ∼460  nm and high intensity in the range of 525 to 680 nm. In the near UV range (up to 430 nm) and the NIR range (beginning at 750 nm), the illumination intensity is very low, which results in a low signal-to-noise ratio (SNR).

### 2.3 Calibration Chain

**Fig. 5:**
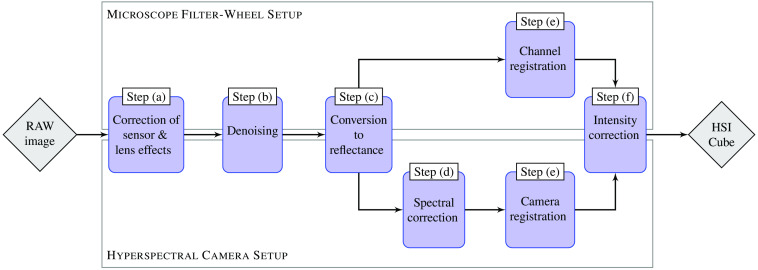
The general calibration workflow for both setups. The calibration steps (a)–(c) as well as (e) and (f) are needed for both setups, whereas step (d) is only performed for the hyperspectral camera setup. Calibration step (a) corrects the internal setup effects of the used sensors and lenses. The sensor noise is reduced in step (b). In step (c), the measured sensor data are converted to physical reflectance values. Step (d) ensures that the specific spectral information is achieved and crosstalks are eliminated. The steps (e) and (f) are necessary for visualization and validation of the spectral behavior of both setups.

The introduced difficulties and impacts on data quality require a robust calibration with reference data. Since the measurement of the reference data includes uncertainties, errors will propagate into the corrected data, which cannot be quantified in the postprocessing. In order to make the acquired data comparable between the two setups and other spectral acquisition techniques, a joint calibration chain is developed in MATLAB, sketched in Fig. 5. The calibration process has to take the following different aspects into account: (a) sensor and lens effect correction, (b) denoising, (c) transforming the measured data to physical units, (d) spectral correction, (e) spectral channel registration, and (f) systematic shift correction. All corrections are performed on the measured sensor data as an entire unit, to also correct effects such as crosstalk.

#### 2.3.1 Sensor and lens effect correction

The camera sensor readout of both setups is larger than the active areas. These inactive regions are omitted in the first calibration step. For the HSI cameras, a correct tile order of the mosaic structure is ensured by an offset correction. Further, to correct the influence of the lens, i.e., vignetting and aperture, as well as the spectral shifts to the corners due to increasing pixel crosstalk, a band-specific correction has to be applied

$$I_{\text{raw}}=\frac{I_{\mathrm{ref}}}{I_{\mathrm{meas}}},$$ (1)

where $I_{\mathrm{meas}}$ is the band-wise measured intensity at every pixel and $I_{\mathrm{ref}}$ is the band-wise averaged intensity in the center of the sensor, with a size of ±10  pixels around the center.

#### 2.3.2 Denoising and conversion to reflectance

The denoising and conversion of raw image data $I_{\text{raw}}$ to reflectance $I_{\mathrm{res}}$ is achieved in one step including white balance alignment and dark current correction as well. A white reference calibration board (95% reflectance Zenith Lite, SphereOptics GmbH, Germany) is scanned with both setups and all illumination settings for the white balance alignment. The achieved intensity $I_{\text{white}}$ allows the conversion of raw image data $I_{\text{raw}}$ to reflectance $I_{\mathrm{res}}$ in a one-point calibration method^26^

$$I_{\mathrm{res}}=\frac{I_{\text{raw}}-(I_{\text{dark}}|\epsilon_{\text{raw}})}{I_{\text{white}}-(I_{\text{dark}}|\epsilon_{\text{white}})}\cdot \frac{\epsilon_{\text{white}}}{\epsilon_{\text{raw}}},$$ (2)

where $I_{\text{raw}}$ is the measured pixel information, $I_{\text{dark}}$ contains the information of a dark reference image (for dark current correction) captured with the corresponding exposure setting ϵ, and the ration of $\frac{\epsilon_{\text{white}}}{\epsilon_{\text{raw}}}$ characterizes the exposure time of the white reference $\epsilon_{\text{white}}$ divided by the exposure time of the measurement $\epsilon_{\text{raw}}$. For the pixel-wise dark current correction, a series of images with the shielded camera-sensor have been acquired and temporally averaged to $I_{\text{dark}}$, as this increases the SNR, defined as

$$\mathrm{SNR}=\frac{A_{\text{signal}}}{\sigma_{\text{noise}}},$$ (3)

with the amplitude of the measured signal $A_{\text{signal}}$ and the standard deviation (SD) of the noise $\sigma_{\text{noise}}$ of the dark current image. To consider changing conditions, $I_{\text{dark}}$ is measured in every measuring sequence using the same setting as the actual acquisitions.

During the calibration, i.e., acquisition of $I_{\text{white}}$, the sensor’s view orientation should be the same as during the measurement and the plane of the calibration board should be parallel to the measured surface. Further, shadows on the calibration board should be avoided and the calibration should be carried out with the same optics as used during measurements. In practical application, this is difficult to realize and small errors will be included in this process as specified already.

#### 2.3.3 Spectral correction

For the hyperspectral camera setup, an additional spectral correction step is needed.^27^ A camera-specific correction matrix C is applied to the calculated reflectance values $I_{\mathrm{res}}$ to achieve the specific spectral information and to eliminate crosstalks of neighboring pixels

$$I_{\mathrm{cor}}=I_{\mathrm{res}}\cdot C,$$ (4)

where the matrix C is specifically computed from the available sensor responses (Fig. 3) using an estimated system model. This matrix C behaves as a deconvolution of the spectral bands. The corrected signal $I_{\mathrm{cor}}$ behaves as if captured with nearly ideal filters. The spectral correction results in 25 channels for the 5×5-sensor and 16 channels for the 4×4-sensor, respectively. The influence of the second-order peaks, see Fig. 3(a), to the measurements are corrected in this step as well. The used spectral channels of both setups are defined in Table 1.

#### 2.3.4 Channel registration

To allow building up the hyperspectral data cube for clinical analysis, both acquired images of the 4×4 and 5×5 HSI cameras have to be spatially registered. Equally, the sequentially acquired filter-wheel images need to be spatially aligned. This registration process uses normalized cross correlation as cost function to register locally specified tissue areas to a hyperspectral data cube.^28^

#### 2.3.5 Intensity correction

Due to uncertainties of the provided calibration information as well as the described internal and external effects, e.g., differences in WD, systematic shifts occur in the reconstructed data for each part in the setups, i.e., filter-wheel setup, 4×4 HSI camera, and 5×5 HSI camera. These identified shifts are corrected in the last calibration step using

$$I_{\text{final}}=\alpha I_{\mathrm{res}},$$ (5)

where the scalar α is the scaling factor and $I_{\mathrm{res}}$ is the reflectance intensity from Eq. (2). This scaling factor α is a proportionality constant, which establishes the correlation between spectral illumination intensity and sensor signal response. For constant WD and sensor, it is sufficient to specify α only once.

**Fig. 6:**
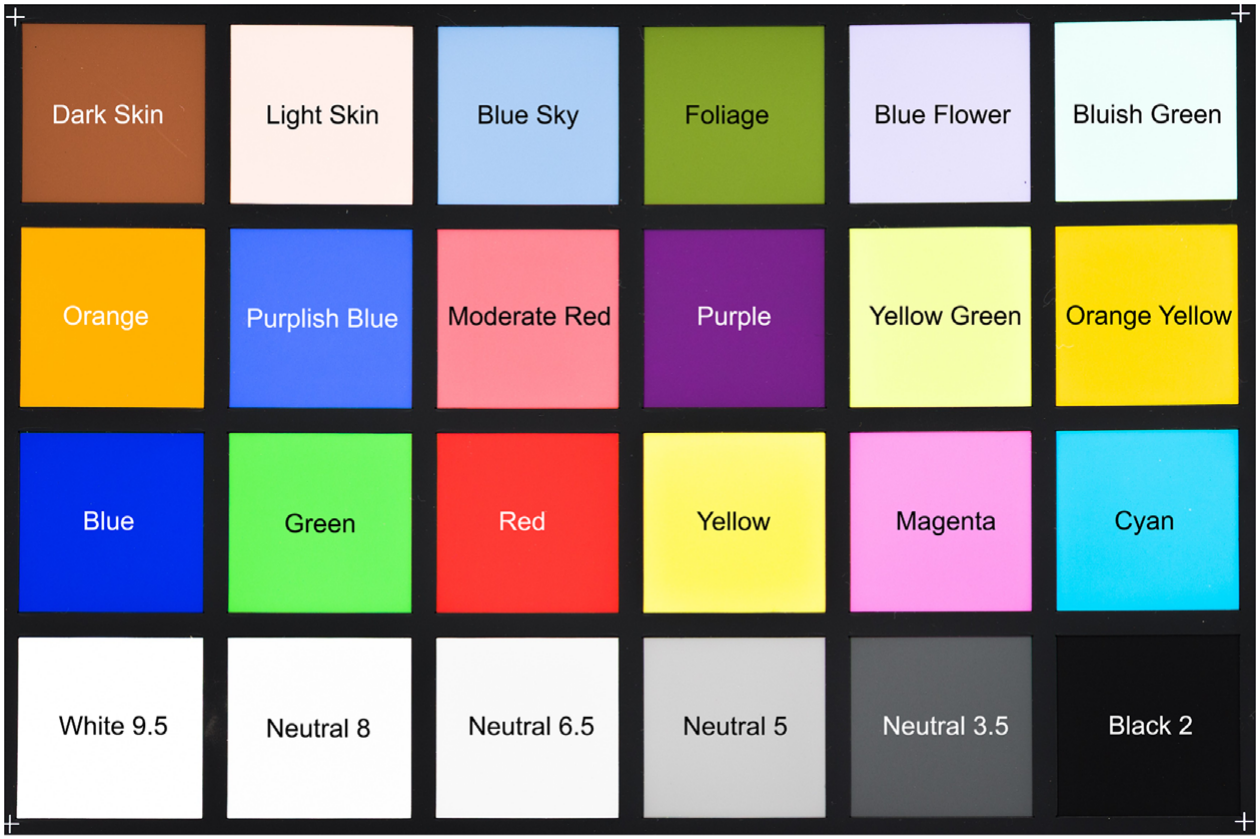
This color chart, ColorChecker Classic, X-Rite, Inc., USA, is used for spectral and scale correction. The 18 colored and 6 gray-scale tiles are used as reference spectra as each color spectrum is well-known. The names of the different colors are written in the corresponding fields to allow a comparison of the measured spectra.

A chart (ColorChecker Classic, X-Rite, Inc., USA) with 18 colored and 6 gray-scale tiles of well-defined color spectra^22^ (see Fig. 6) is scanned with both setups and both illumination options and analyzed to identify α and correct the spectral information assuring an accurate comparison between both approaches. Each color spectrum is known in terms of reflectance, with SD for each data point, in the range of 390 and 1000 nm with a step size of 10 nm. This information is used as reference for the scaling calibration and generated using the broadband spectroradiometer specbos 1211UV-2, JETI Technische Instrumente GmbH, Germany. The respective SD over all reference measurements varies between all wavelengths and all color tiles, which is consistent with the literature.^29^ It lies between 0.2%, i.e., 0.002, (e.g., for tile “foliage” in the range of 430 to 460 nm, or for tile “blue” in the range of 590 to 630 nm) up to 11.0%, i.e., 0.110, (e.g., for tile “light skin” 710 to 730 nm, or tile “purple” 720 to 730 nm). Some color tiles, e.g. “white 9.5,” “light skin,” “bluish green,” “blue,” “orange yellow,” or “yellow,” show larger SD over the whole spectral range, whereas other colors (e.g., “dark skin,” “cyan,” or “magenta”) have only small SD.

The presented calibration chain does not take into account thermal effects or scattered light, thus these need to be avoided as described in Sec. 2.1. To allow comparison of measured clinical data, acquired with both setups, also the magnification has to be comparable. This analysis is done using a checkerboard allowing the comparison of the segmentation results.

### 2.4 First Clinical in vivo Measurements

To prove both presented approaches in the clinical environment, we have acquired first clinical *in vivo* data. The measurements have taken place during two otorhinolaryngology surgeries. One measurement has been performed during a neck dissection and another during a parotidectomy. In both cases, the surgeon annotated the individual tissue types for later analysis. The HSI camera setup uses two snapshot cameras that both are focusing on the same situs area with a slightly different angle. In both of the resulting images, the surgeon manually annotates the tissue types of interest. Thereby, the surgeon tags areas that cover exactly the same anatomical regions in both images and the two reconstructed spectral information can be combined to one full spectrum. The regions of different tissue types have been analyzed individually to reconstruct the specific spectral behavior of each type.

For the visualization of interesting spectral behaviors, the spectral data are enhanced selecting relevant spectral intervals by a weighting matrix W with size 41×41 due to 41 λ-bands and the principal component analysis (PCA)^30^ using

$$J_{\text{enhanced}}=W\overset{N}{\underset{i=m+1}{\sum }}\gamma_{i}A_{i},$$ (6)

where $A_{i}$ is the i’th PCA basis vector, $\gamma_{i}$ is a coefficient of the specific PCA component, and W holds the magnification of the specific selected λs.^31^^,^^32^ The enhanced image data are added to the blue channel of the calculated RGB image.^33^ The whole capturing procedure is in agreement with the ethical approval obtained from the Ethics Committee of the Charité—Universitätsmedizin Berlin.

## 3 Results

### 3.1 Calibration Results

#### 3.1.1 Magnification

The magnification of the camera’s field of view differs for both setups. The difference between the filter-wheel and the hyperspectral camera setup is ∼3:1. The distances between the optics of each setup and the object are identical and in the filter-wheel setup the highest zoom-level is set. This has to be considered during postprocessing and tissue behavior analysis, but has no impact on the quantitative results of this work.

#### 3.1.2 Calibration chain

**Fig. 7:**
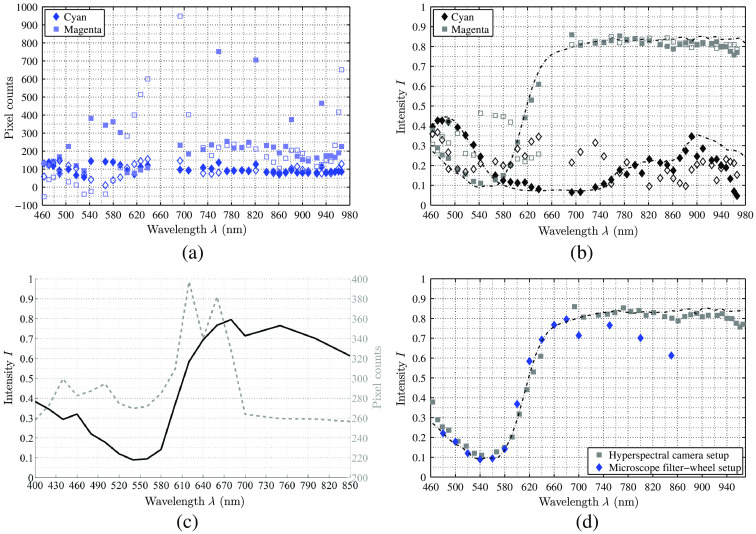
These curves substantiate the need of the described calibration chain. (a), (b) The two reference spectra (□, magenta tile and ⋄, cyan tile) of the hyperspectral camera setup. (a) The raw data, the filled markers represent the raw sensor counts, and the unfilled markers represent the sensor counts corrected with the specific correction matrix C from Eq. (4). (b) The corrected and white balanced data (the dotted lines show the reference data of the color chart). The unfilled markers represent the calculated reflectance data $I_{\mathrm{res}}$ using Eq. (2) without applying Eq. (4) and the filled markers represent the fully corrected data $I_{\text{final}}$, which is the fully corrected physical reflectance. For the microscope filter-wheel setup, (c) shows the same behavior of the “magenta” tile. (d) The pixel counts (dotted line) are completely different to the HSI camera setup but the reconstructed spectrum (black line) behaves as the reconstructed line of the HSI camera setup and the reference data, which shows the reconstructed magenta spectrum of both setups with the specific reference data of the color chart as dotted line.

Figure 7 substantiate the need of the described calibration chain. It shows that acquired pixel values do not represent the actual reflectance information and highly differ between different setups. The comparison of Figs. 7(a) and 7(b) shows that only the combination of spectral calibration and conversion to reflection will result in a physically correct spectrum.

For the two different illumination possibilities of the HSI camera setup, the exposure time is longer with Xe illumination, as the luminous flux of the surgical LED light is much higher, cf., Sec. 2.2. The exposure time must be selected such that the full capacity of the sensor sensitivity is optimally used for all bands while a saturation of single bands is avoided. Hence, some channels do not use the full sensor sensitivity and the calculated $I_{\mathrm{res}}$ could be underestimated. To use the full capacity of the sensor sensitivity, the exposure time for the 4×4 HSI camera is lower compared to the 5×5 HSI camera, resulting in a lower frame rate for the 5×5 HSI camera. This is caused by the differences in the sensor response, cf., Fig. 3, as well as the differences in the illumination intensity through the different wavelengths. A low frame rate has no effect on the calibration results, but has to be considered for clinical measurements. Thus for the 5×5 HSI camera, an exposure time is chosen, which does not use the full sensor sensitivity but keeps an acceptable frame rate. This results in a statistical bias. Therefore, the introduced calibration step (f) intensity correction is essential for accurate and comparable signal processing. It will correct such underestimations as well as differences in WD between the individual cameras using the reference curves. The optimal exposure times identified for our setups and illumination settings are presented in Table 2.

**Table 2:** The identified optimal exposure times per frame for our two setups. For the filter-wheel setup, the stated exposure time is per wavelength band.

Setup	Filter-wheel	4×4 camera	4×4 camera	5×5 camera	5×5 camera
Illumination	Xe	Xe	LED	Xe	LED
Exposure time (ms)	42	65	0.5	110	7.5

The characteristics of the reference color spectra have been correctly reconstructed for all three settings (microscope filter-wheel setup and hyperspectral camera setup with two illumination possibilities) using the proposed calibration steps (a)–(e). A systematic scale shift is observed in the reconstructed spectral points for both setups. This shift depends on the camera type, the illumination setting and distance as well as the WD. Calibration step (f) corrects most of these effects. A weighting factor α is applied, according to the different systematic behaviors of the different setups, see Table 3.

**Table 3:** The identified weighting factors α to correct systematic scale shifts of the setup calibration.

Setup	Filter-wheel	4×4 camera	4×4 camera	5×5 camera	5×5 camera
Illumination	Xe	Xe	LED	Xe	LED
α	2.01	1.00	1.07	1.22	1.03

The filter-wheel setup shows a clearly visible systematic shift, which has to be compensated with α=2.01. In the NIR, this shift is growing due to illumination inhomogeneities and a small SNR. The growing part of this shift will not corrected by α, cf., Fig. 12. For the 4×4 HSI camera, the internal and external effects, e.g., WD and illumination setting, compensate each other which results in no (α=1.00) or no significant (α=1.07) difference for the Xe and LED illumination, respectively. The same occurs for the 5×5 HSI camera with LED illumination (α=1.03). Due to the above-mentioned challenges with very low SNR as well as the high deviation of the reference data and measurements in NIR, the values above λ>850  nm are neglected for α-optimization. For the 5×5 HSI camera with Xe illumination, the shift is larger with α=1.22.

**Fig. 8:**
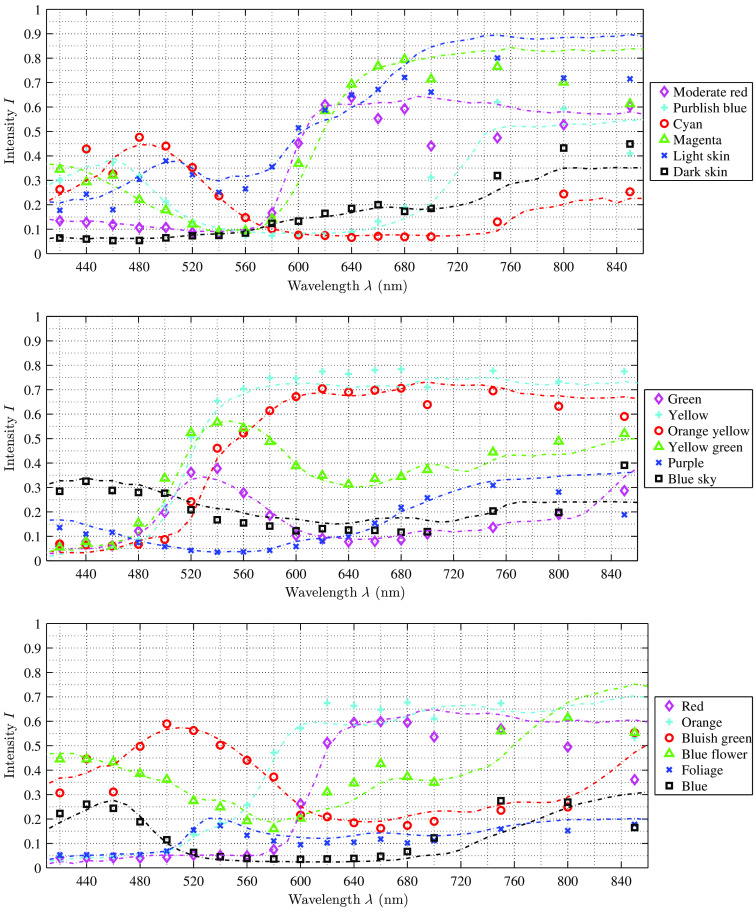
Calibration results of all analyzed color spectra with the filter-wheel setup. The reference data, plotted as dotted lines, could be fully reconstructed for all curves. The reconstructed data points have a distance of 20 nm in the visual range and a distance of 50 nm in the NIR. The results are comparable to the results of the hyperspectral camera setup (Figs. 9 and 10).

**Fig. 9:**
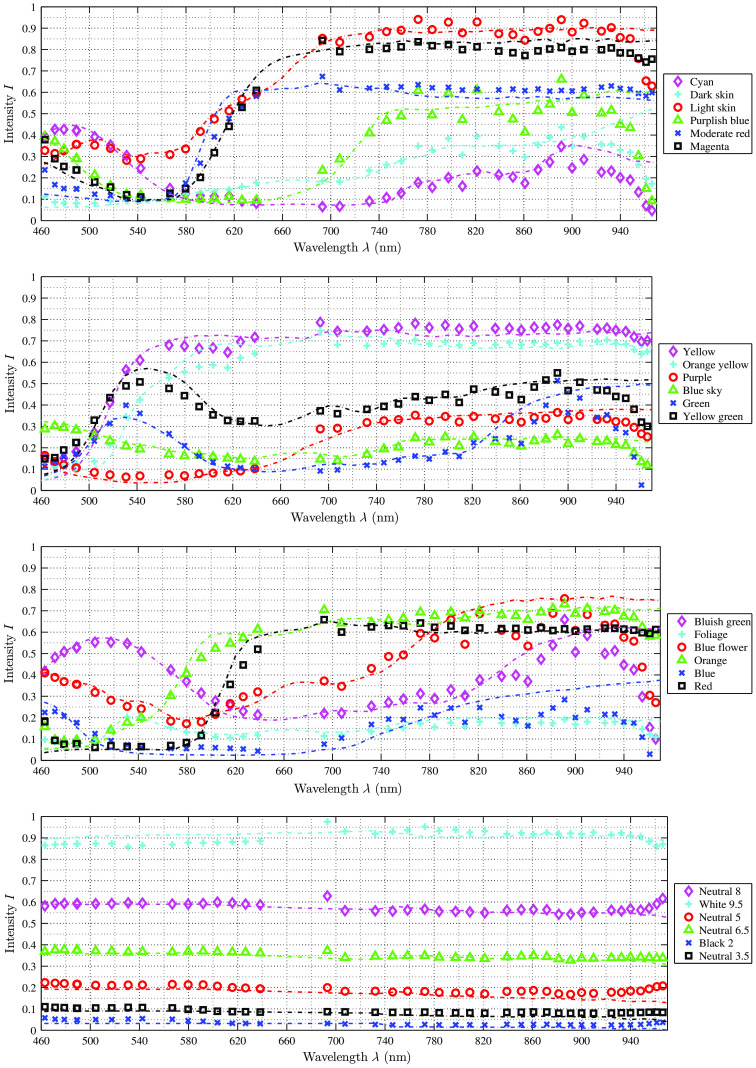
Calibration results of all 24 analyzed color spectra with the hyperspectral snapshot camera setup and Xe illumination. The reference data, plotted as dotted lines, could be fully reconstructed for all curves. In the region of NIR (λ>840  nm), the deviation d between reconstruction and reference is increasing, but the results are comparable to the results of the microscope filter-wheel setup (Fig. 8) as well as the HSI camera setup with LED illumination, see Fig. 10.

**Fig. 10:**
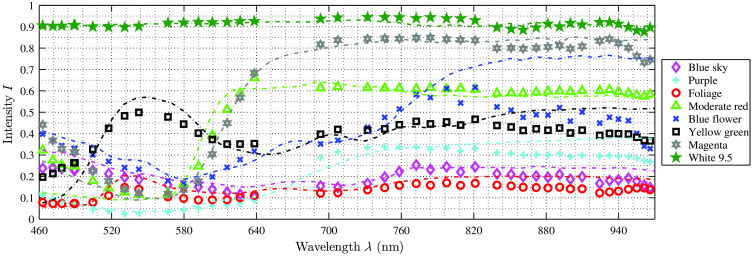
A selection of eight out of the 24 measured reference spectra acquired with the hyperspectral camera setup used with LED surgical light. The reference information is plotted as dotted lines. The results show the same behavior for all scanned spectra as with the Xe illumination setup, cf., Fig. 9.

Figure 8 plots all 18 reconstructed color spectra, acquired with the filter-wheel setup and corrected with the identified weighting factor α. The data fits to the reference spectra (of the color chart—dotted lines in Figs. 8–10). All reference data are within the calculated SD of the reconstructed data points as well as all reconstructed data points are within the respective SD of the reference color board. For all color tiles of the chart, the deviation between reference and calculated intensity increases starting at ∼680  nm, due to the following reasons: First, the SD of the reference spectra is increasing at about λ>650  nm. In the visual range (420 to 680 nm), the SD is below 1%, while for the red and NIR spectrum it increases from 2% up to 11%. Second, for the NIR range, the images contain low signal intensities and the SNR is decreasing. The SNR in the visual range is SNR>3 and SNR≈2 at 700 nm, whereas at 400, 750, and 800 nm, it is SNR≈1 and for 850 nm it is decreasing to SNR≈0.1.

Figure 9 shows the 18 reconstructed and corrected color spectra and six different gray scales with its respective reference spectrum for the hyperspectral camera setup and Xe illumination. The plots demonstrate that the reconstructed data of the 4×4 camera (460  nm<λ<650  nm) fit the reference values very well. All differences d between reconstructed and reference intensities are within the permissible error tolerances (measured SD as well as stated reference SD). The reconstructed data of the 5×5 camera show higher differences d between reconstructed and reference intensities, especially for the reconstructed data at about λ>840  nm. With increasing λ>840  nm, the difference d increases. This effect has several reasons. As described above, the SD of the measured and reference data is increasing in the NIR range, whereas the SNR is very low in that spectral range. Thus it becomes complicated to reconstruct the signal. Further, the large spreading of the highest intensity value between the individual bands on the 5×5 HSI sensor [cf., Fig. 3(b)] results in the behavior that some bands do not use the full sensor sensitivity while other bands could reach saturation. This explains the high variability for the last six data points (λ>930  nm) as well as the increased deviation of the first data point (λ=693  nm), since the sensor response is very low for these bands. The same effect occurs at λ=400  nm for the filter-wheel setup as the light sources are clipping at λ=400  nm to avoid UV illumination. The same behavior, described for the HSI camera and Xe illumination, is observed for the HSI camera with LED illumination. Figure 10 shows a selection of eight reconstructed color spectra.

**Fig. 11:**
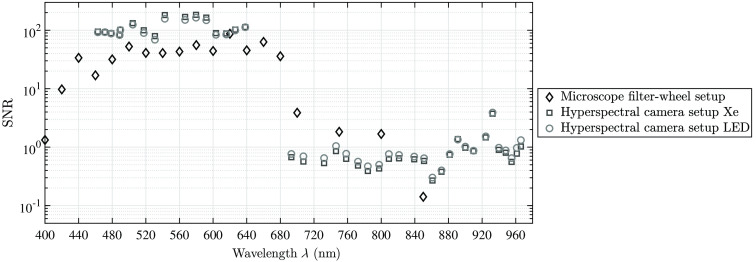
The average wavelength-dependent SNR values of each setup. In the visual range, the HSI camera setup shows an increased SNR for both illumination settings compared to the microscopic filter-wheel setup. In the NIR range, the general SNR is much lower. The microscope setup and the HSI camera setup with LED illumination show similar SNR, while for the HSI camera setup with Xe illumination, the SNR is even smaller in the range of 680 to 880 nm.

Compared to the filter-wheel setup, the SNR is higher at each measured wavelength in the visual range and slightly smaller in the NIR, except for λ=850  nm where the SNR is higher. For Xe illumination, the average SNR for the visual range (4×4 camera) is ${\mathrm{SNR}}_{\mathrm{avg}}=116.55$ (${\mathrm{SNR}}_{\min}=6.19$ and ${\mathrm{SNR}}_{\max}=394.45$) and for the NIR range (5×5 camera) ${\mathrm{SNR}}_{\mathrm{avg}}=0.84$. For the LED illumination, the average SNR for the visual range is ${\mathrm{SNR}}_{\mathrm{avg}}=108.68$ (${\mathrm{SNR}}_{\min}=3.57$ and ${\mathrm{SNR}}_{\max}=413.36$) and for the NIR range ${\mathrm{SNR}}_{\mathrm{avg}}=0.93$ (${\mathrm{SNR}}_{\min}=0.02$ and ${\mathrm{SNR}}_{\max}=9.19$). As can be seen in Fig. 11, the SNR between both illumination options is similar. However, it depends on the chosen exposure time and the used light source. Since the luminous flux of the surgical LED light is much higher compared to the Xe source, the SNR is increased in the NIR using LED illumination. Overall, Fig. 11 shows that the SNR is low for the NIR due to several different effects. The illumination intensity decreases for the LED source in the NIR, which results in a lower light reception on the sensor. Additionally, the filter properties of the 5×5 snapshot camera play an important role as stated above, cf., Fig. 3(b). In the spectral range of 680 to 850 nm, the SNR is relatively homogenous between 0.6≤SNR≤0.8 and shows larger fluctuation for λ>885  nm. This behavior corresponds to the different peak intensities of the individual bands. The bands with very low intensity of $I_{\text{peak}}\approx 0.04$ are related to the wavelengths with very small SNR.

**Fig. 12:**
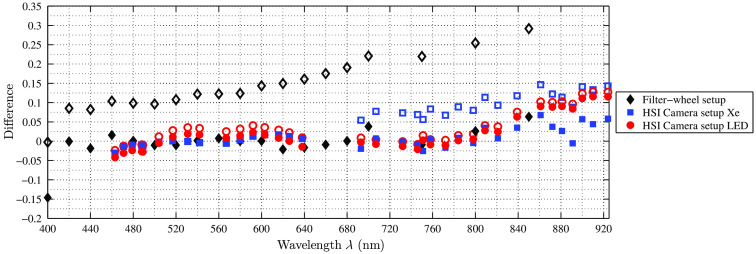
The quality of the complete calibration chain. The unfilled markers show the calibrated setups without the correction factor α. In this case, the filter-wheel setup shows a systematic shift. Due to the smaller SNR of the NIR data, the gap d between reference and reconstructed data (“difference”—plotted on the y axis) is increasing for all setups. The filled markers show that a correction factor α can adjust all settings to the reference and allows comparison among the setup data.

To compare all three introduced settings, the difference d between the reconstructed spectral point to its reference value has been calculated for each setting. Figure 12 shows that the variances from the reference values are reduced when the scale correction using the presented α of Table 3 is applied. The unfilled markers in Fig. 12 show the gap between the reference data and the reconstructed data using calibration steps (a)–(e), whereas the filled markers represent the results applying the complete calibration pipeline (a)–(f). In this case, all data points are around d=0, except for the data of λ>850  nm due to the described uncertainties. Hence, the measured data of all three settings become comparable using the calibration pipeline introduced here and the setups can be used for optical tissue analysis, as shown in the next section.

### 3.2 First Clinical in vivo Measurements

**Fig. 13:**
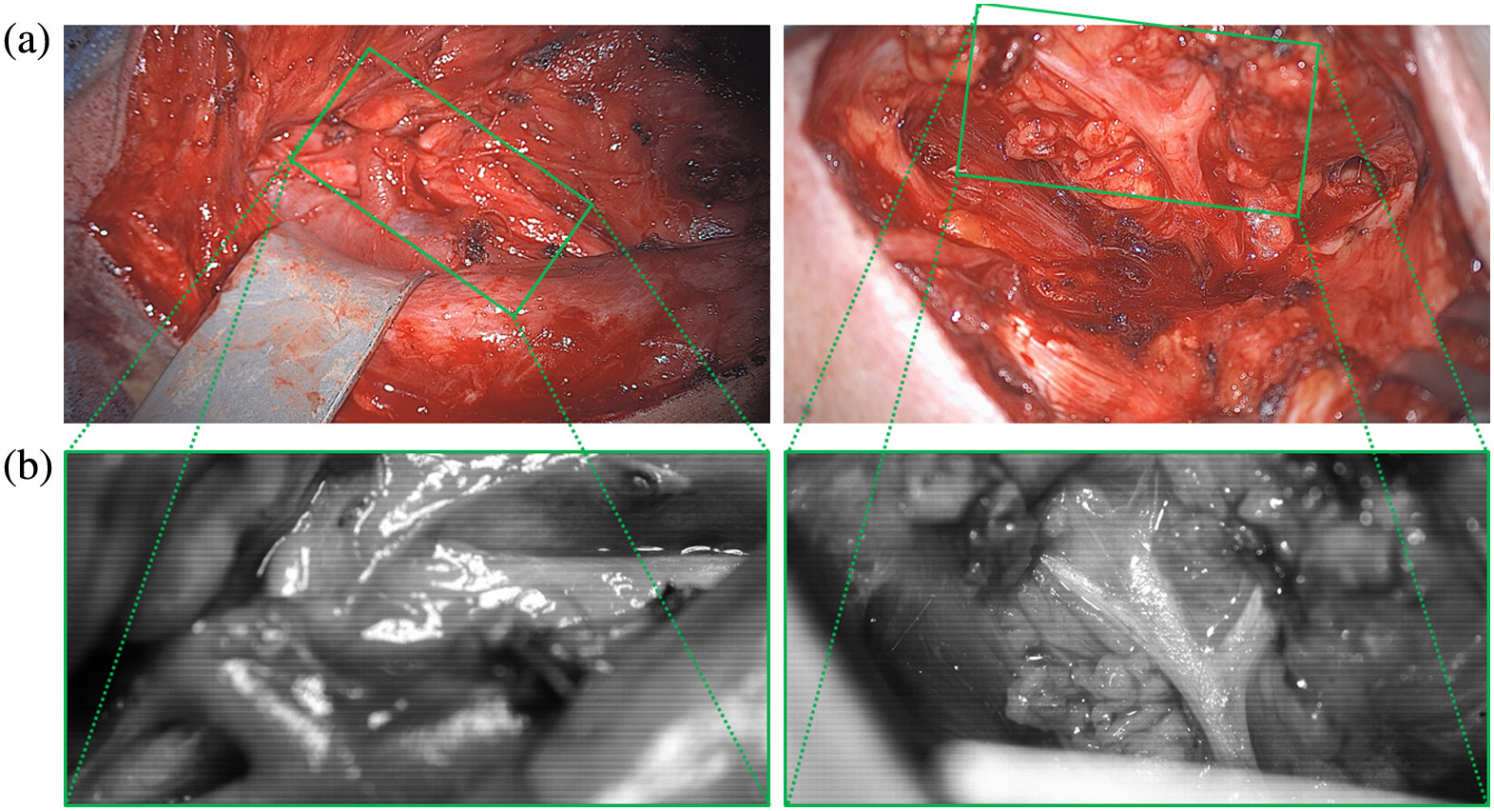
The area of the situs that has been scanned with our setups. (a) The complete situs of the two surgeries and (b) the two views measured with the 4×4 HSI camera, with its sketched location in the situs. The camera positions of the RGB overview image and HSI image are not identical, which produces projection differences.

**Fig. 14:**
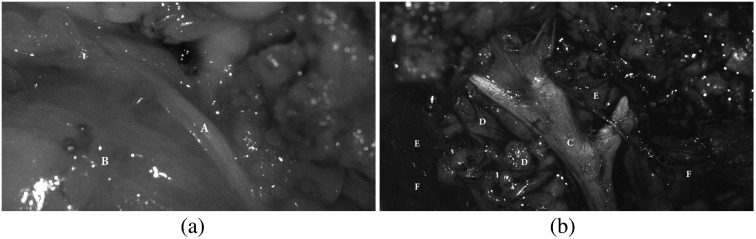
Two analyzed views of the two patients measured with the filter-wheel setup. (a) The image acquired with illumination at λ=680  nm and (b) the image acquired with λ=460  nm. The interesting tissues are labeled as A, artery; B, vein; C, nerv; D, parotid gland; E, connective tissue; and F, muscle.

**Fig. 15:**
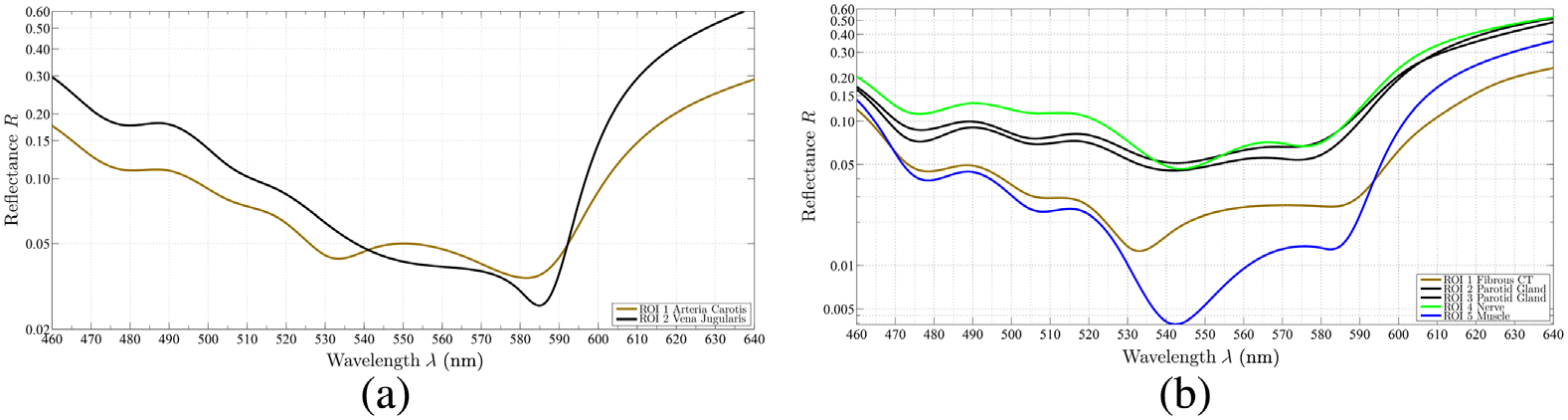
The spectra of several analyzed *in vivo* tissue behaviors in the visual range. (a) The reflectance of vein and artery of patient 1 and (b) the reflectance behavior of muscle, parotid gland, nerve, and fibrous connective tissue. All tissue types show different optical behaviors through the analyzed spectrum.

We have scanned the situs with our setups during two surgical standard procedures. Figure 13(a) shows the situs of both surgeries (patient 1 on the left and patient 2 on the right). In Fig. 13(b), the view to the situs of the 4×4 snapshot camera is shown. The view of the 5×5 snapshot cameras appears in a similar way with less intensity. The microscope filter-wheel setup has been located right next to the snapshot cameras. Figure 14 shows two measured views of two different patients with the filter-wheel setup, from which the optical behaviors of different tissue types are extracted. Compared to the views of the snapshot cameras, the scene is shifted and in the first case additionally rotated. The left image of patient 1, the neck dissection, shows the scene at λ=680  nm illumination, whereas the right image of patient 2, the parotidectomy, shows the scene at λ=460  nm illumination. Although both views are from different patients, these images show that different tissue types show different optical behaviors under different wavelength illuminations, see also Fig. 15. Clearly visible is this fact in Fig. 13, as the mosaic pattern with all 16 visual wavelength bands is visible and these pattern behave differently for different tissue types.

Our first two clinical *in vivo* measurements of human soft tissue show different visible behaviors between the various wavelengths for different tissue types. After the surgeon annotated the tissue types in the captured images, binary masks are created to evaluate each type independently and reconstruct the physical reflectance R, shown in Fig. 15. As the visible range (460 to 640 nm) is of highest interest for intraoperative visualization, this interval of the reconstructed spectra is presented in Fig. 15. In the data of patient 1 [Fig. 15(a)], the neck dissection, the focus of the analysis is on the blood vessels, as artery (in this case: arteria carotis) and vein (vena jugularis) transport blood with oxygenated and deoxygenated hemoglobin (Hb), respectively. The analyzed spectral behavior of captured arteria carotis and vena jugularis correspond to the optical properties of human blood presented in the literature.^34^^,^^35^ The reflectance for oxygenated Hb (arterial blood) is higher in the range of 540 to 590 nm and in the other parts of the visual spectrum lower than the reflectance for deoxygenated Hb (venous blood). Further, oxygenated Hb shows the two characteristic minima in the range of 530 to 585 nm. For the data of patient 2, the parotidectomy, the focus of the analysis is on several different soft tissue types present in the situs: muscle, nerve, parotid gland, and fibrous connective tissue. The visible range of the reconstructed spectra is shown in Fig. 15(b). These spectral data correspond to *in vivo* measurements using a spectrophotometer.^36^^,^^37^ Nervous tissue reflects with the highest intensity in the spectral range λ<530  nm. In the range λ>530  nm, it is similar to parotid gland tissue. The spectral behaviors of muscle and fibrous connective tissue are lower in terms of reflectance compared to nerve and parotid gland over the whole analyzed spectrum. Until λ=530  nm, both tissues show similar reflectance behavior, while starting at λ>530  nm high differences exist between both tissues. These tissue differences in its spectral behaviors indicate that this knowledge can be used to allow intraoperative tissue differentiation.

Both introduced setups, microscopic filter-wheel as well as snapshot cameras, reveal the same tissue characteristics in both cases with respect to internal and external uncertainties. Since a 3D tissue structure is captured, the surface is never perfectly aligned to the camera and different tissue types have different orientations to the sensor. Therefore, the reconstructed optical behavior can differ in a single tissue structure if it appears at different areas in the image, which results in slightly different spectra for a single tissue type. This is shown in Fig. 15(b) for parotid gland curves (black curves). Both curves represent the same tissue type present in different areas in the image (with slightly different WD and other variances), resulting in a small deviation of both curves but showing the same overall characteristics.

**Fig. 16:**
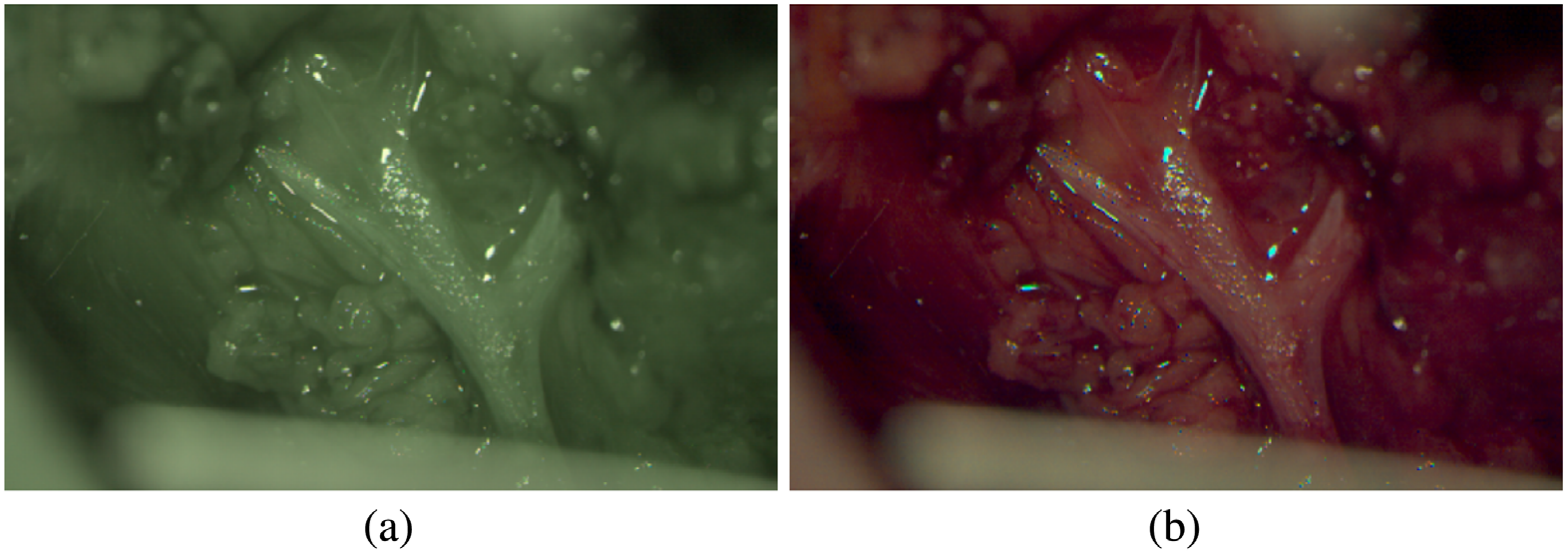
The reconstructed RGB calculation using the same measured hyperspectral snapshot data and the CIE color matching functions. The measured raw pixel counts are used for RGB calculation in (a) which results in a false greenish RGB appearance, whereas in view (b) the corrected reconstructed spectral data are used for RGB calculation, which results in a realistic RGB appearance.

**Fig. 17:**
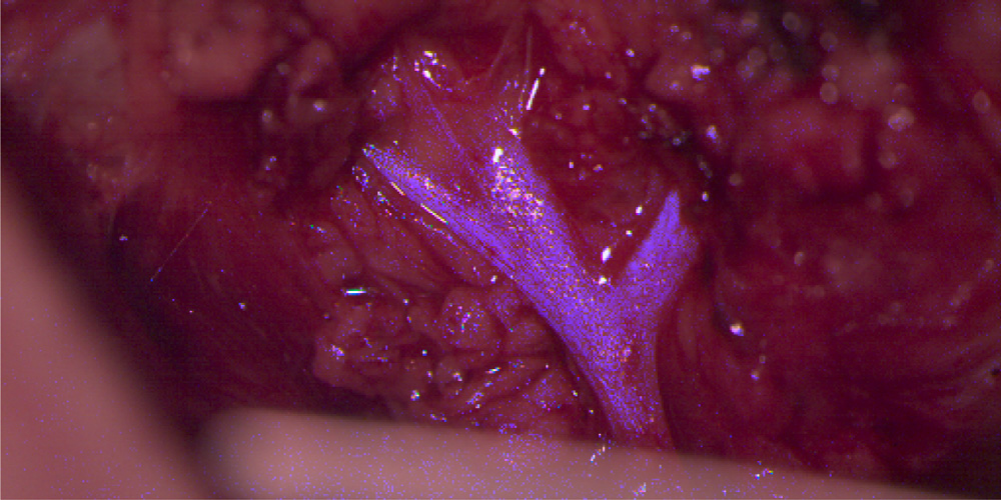
The same reconstructed RGB-view as presented in Fig. 16 but with enhanced nerve visualization. In this case, after RGB calculation of the calibrated multispectral data using the CIE color matching functions, the resulted nerve enhancement is added to the B-channel, which results in a nerve enhanced RGB representation.

For intraoperative usage, a reasonable visualization of the information extracted from the hyperspectral data is needed. A classical RGB image can be calculated from the hyperspectral data using the CIE color matching functions. Figure 16 shows the reconstructed RGB image from the raw pixel counts for every measured λ as well as from the calibrated reflectance data, illustrating the need of the presented calibration pipeline. Without calibration, the wrong spectrum [cf., Fig. 7(c)] results in an overweighting of the green image channel, Fig. 16(a), while the presented calibration pipeline results in a realistic RGB appearance of the scene as it would appear in a conventional surgical microscope, Fig. 16(b). Such an RGB calculation from multispectral data is done at the expense of information loss, since the specific multispectral information is combined in three channels (RGB). Thus the different reflectance properties over the complete analyzed spectrum of the individual tissue types have to be used in an additional step to enhance interesting structures in the calculated RGB image. For example, during otorhinolaryngology surgeries the facial nerve as tissue of risk has to be completely preserved. Therefore in parotidectomy, nerve preparation requires most of the surgical time and is an extremely complex task, as the facial nerve splits into five delicate branches performing important functions as, e.g., facial expression and sensation. Thus the facial nerve should not be injured in any way and a better visualization of the nerve would be beneficial for the preparation as well as the complete surgical process keeping a constant watch on the nerve. To demonstrate a possible application of the presented approach in an intraoperative assistance system, the measured reflectance properties of patient 2 are used to highlight the nervous tissue in the finally calculated RGB visualization, see Fig. 17. The measured data in the spectral range of 460 to 480 nm are enhanced using knowledge about the general optical tissue behaviors^37^ and the described PCA method of Eq. (6). Spectral differences between image regions, i.e., different tissue types, in the range of 460 to 480 nm are enlarged using an enhancement factor. This enhanced differences are then added to the blue channel of the calculated RGB image [cf., Fig. 16(b)] resulting in a blue appearance of nervous tissue structures (cf., Fig. 17). Using this image, the surgeon is able to identify the nerve easily and monitor it during the entire surgical procedure.

## 4 Conclusion

In this work, two different hyperspectral setups are presented and calibrated for intraoperative tissue analysis. Both setups perform the wavelength selection in different parts of the processing chain, complicating the comparison of the results. The proposed calibration pipeline makes a comparison between both approaches possible and aligns the resulting data such that the same tissue analysis methods can be applied. The microscope filter-wheel setup uses monochromatic illumination of the scene with a camera sensitive over the complete spectral range (400 to 850 nm). In the hyperspectral camera setup, the scene is illuminated with white light and separation of the spectral response is done through the sensor setup (with a spectral range of 463 up to 966 nm).

In the visual range, the SNR is very high, while it decreases in the NIR. We propose to use a band-adapted exposure time with the hyperspectral camera setup to address the low SNR in NIR, which would require several frame acquisitions. Due to increased exposure time, some bands will reach saturation (e.g., bands 5 to 14 on the 5×5 sensor, cf., Fig. 3), whereas the SNR of other bands (e.g., bands 0 to 4 and 15 to 24 on the 5×5 sensor) will be increased to a better level. Appropriate bands are then selected from the respective frames.

The reconstructed reflection data of both setups show the same quality compared to the reference data after using our proposed calibration chain, cf., Fig. 7(d). This allows a comparison of the measured data between the different setups and to other acquisition techniques as spectrometry. Such a robust calibration is essential for soft tissue analysis, as these tissue types show large variability. Further, both setups have been successfully employed in the clinical environment for two different ENT surgeries as at the head region many important tissue structures are at close range. It is shown that these hyperspectral setups with the introduced calibration have the potential to present enhanced relevant tissues as nervous tissue during surgery. Based on our calibration and preliminary results, further studies of optical tissue behavior, especially human soft tissue, can take place with both setups.

In summary, this study shows that both introduced HSI modalities are suitable for intraoperative HSI and the proposed calibration pipeline makes the data comparable between both approaches, but also to other external tissue analysis modalities, such as soft tissue analysis using spectrophotometry.^36^^–^^38^ Thus the two imaging modalities can be used alternately depending on the requirements and acceptance of the clinical needs and requirements. A modified microscope setup could be used during microscopic or endoscopic surgeries, whereas a hyperspectral camera setup additionally could be used during conventional open surgeries.
